# Is There an Optimal Timing of Autologous Stem-Cell Transplantation for Multiple Myeloma in the Era of Novel Agents?

**DOI:** 10.4274/Tjh.2013.0079

**Published:** 2013-09-05

**Authors:** Selami Koçak Toprak

**Affiliations:** 1 Başkent University School of Medicine, Department of Hematology, Ankara, Turkey

## TO THE EDITOR

In the last couple of years, parallel to the developments in our knowledge of disease pathogenesis, the inclusion of immunomodulatory drugs like thalidomide and lenalidomide and various protease inhibitors such as bortezomib to treatment protocols in clinical practice has led to dramatic changes in the treatment of multiple myeloma (MM). These agents particularly have survival advantages in relapse/refractory cases, while changing the classic first-line treatment approach with their recent good results in frontline treatment [1]. The performance of high-dose therapy plus autologous stem cell transplantation (HDT-ASCT) as soon as possible in eligible patients has been the standard treatment approach for almost 20 years [2]. In this period, in many regions, including Turkey, the performance of generally 3 to 6 cycles of an induction treatment consisting of vincristine–doxorubicin–dexamethasone (VAD) with the aim of improving hematopoietic cell collection by firstly decreasing plasma cell infiltration and reducing the tumor burden and increasing post-transplantation complete response rates has been widely done. However, the pre-transplantation results of this treatment have always been disappointing [1]. Nevertheless, the pre- and post-transplantation response rates of a treatment combination like thalidomide–bortezomib–dexamethasone (VTD) have been prominently superior, while the presence of bad prognosis markers, especially t(4;14), del(13q), and del(17p), requires the use of new agents [3]. Supporting this, a study by Palumbo et al. revealed that induction combinations including bortezomib and consequent post-transplantation consolidative-maintenance lenalidomide have led to a complete response (CR) in 66% of cases [4].

In the era of such good results in pre-transplantation treatment with new agents, should the HDT-ASCT approach be used in every patient as soon as possible? Or should it absolutely be performed? Depending on the increased rate of CR and prolonged progression-free survival (PFS) when compared with conventional chemotherapy in several randomized phase III studies, HDT-ASCT has been considered the standard of care for eligible patients with newly diagnosed MM [5]. However, HDT-ASCT is not curative and progression/relapses occur in most patients. Furthermore, according to a systematic review and meta-analysis published in 2007, single HDT-ASCT was not superior to conventional first-line treatments in terms of total survival; additionally, rates of treatment-related mortality were also higher [6]. Results of phase III studies have proven that combinations of new agents like VTD, bortezomib–doxorubicin–dexamethasone, lenalidomide with low-dose dexamethasone (Rd), bortezomib–dexamethasone, thalidomide–dexamethasone, and low-dose bortezomib–thalidomide–dexamethasone are superior to conventional approaches when used in first-line treatment [7]. The high overall and CR rates obtained with new agents have led to suspicions regarding the role and timing of transplantation in the minds of all clinicians, especially in the United States (US); in the US and many other countries, the idea has developed that first-line induction treatment with new agents should be prolonged until relapse/progress -with the condition that stem cells are collected in this period- and, thus, that transplantation should be left for relapse. In this regard, Rajkumar suggested the risk-adopted approach in the selection of first-line treatment for newly diagnosed MM patients and indicated that in standard-risk disease (about 75% of all cases), first-line treatment should consist of Rd × 4 cycles (stem cells would be collected during this period), followed by early or delayed HDT-ASCT performed in accordance with the patient’s choice [8]. Intermediate-risk patients, who are 10% of the population, should receive bortezomib-based first-line treatment followed by early HDT-ASCT and then again bortezomib-based maintenance treatment. In the remaining 15% who have high-risk disease, induction with bortezomib and lenalidomide-based combination should be followed by HDT-ASCT and then bortezomib-based maintenance treatment; however, it was pointed out that in this patient group with a median survival of 2-3 years, clinical studies about more efficient treatments are required [8,9]. In a prior study, new agents were used and patients were evaluated in 2 groups according to HDT-ASCT performed in the first year or later; in the group with induction treatment consisting of lenalidomide–dexamethasone, 4-year survival was over 80%, independently of the timing of transplantation [10]. Nevertheless, in the phase III study of Boccadoro et al., new agents were compared with HDT-ASCT in 402 newly diagnosed MM patients and total survival was not different between 2 arms, while PFS was significantly higher in the arm with autologous stem cell transplantation [11]. As a result, despite prominently superior results of first-line treatment with combinations consisting of new agents, followed by HDT-ASCT and then by maintenance treatment with new agents, in order to decide to exclude HDT-ASCT and perform first-line induction treatment with new agents until relapse, results of multi-center randomized studies performed on large patient groups should be awaited.

## CONFLICT OF INTEREST STATEMENT

The authors of this paper have no conflicts of interest, including specific financial interests, relationships, and/ or affiliations relevant to the subject matter or materials included. 
